# A Retrospective Survey Comparing Suture Techniques Regarding the Risk of Permanent Epicardial Pacemaker Implantation After Ventricular Septal Defect Closure

**DOI:** 10.21470/1678-9741-2018-0010

**Published:** 2018

**Authors:** Mehmet Fatih Ayık, Emrah Şişli, Münevver Dereli, Yasemin Özdemir Şahan, Hatice Şahin, Reşit Ertürk Levent, Yüksel Atay

**Affiliations:** 1 Department of Cardiovascular Surgery, Faculty of Medicine, Ege University, Izmir, Turkey.; 2 Pediatric Cardiology, Faculty of Medicine, Ege University, Izmir, Turkey.; 3 Medical Education, Faculty of Medicine, Ege University, Izmir, Turkey.

**Keywords:** Heart Defects, Congenital, Heart Septal Defects, Ventricular, Suture Technique, Heart Block, Pacemaker, Artificial

## Abstract

**Objective:**

The aim of this study is to compare the continuous and combined suturing techniques in regards to the needing epicardial pacing at the time of weaning from cardiopulmonary bypass (EP-CPB) and to evaluate permanent epicardial pacemaker (PEP) implantation in patients who had undergone surgical ventricular septal defect (VSD) closure.

**Methods:**

This single-centre retrospective survey includes 365 patients who had consecutively undergone VSD closure between January 2006 and October 2015.

**Results:**

The median age and weight of the patients were 15 months (range 27 days - 56.9 years) and 10 kg (range 3.5 - 100 kg), respectively. Continuous and combined suturing techniques were utilised in 302 (82.7%) and 63 (17.3%) patients, respectively. While 25 (6.8%) patients required EP-CPB, PEP was implanted in eight (2.2%) patients. Comparison of the continuous and combined suturing techniques regarding the need for EP-CPB (72% *vs.* 28%, *P*=0.231) and PEP implantation (87.5% *vs*. 12.5%, *P*=1.0) were not statistically significant. The rate of PEP implantation in patients with perimembraneous VSD without extension and perimembraneous VSD with inlet extension did not reveal significant difference between the suture techniques (*P*=1.0 and *P*=0.16, respectively). In both univariate and multivariate analyses, large VSD (*P*=0.001; OR 8.63; *P*=0.011) and perimembraneous VSD with inlet extension (*P*<0.001; OR 9.02; *P*=0.005) had a significant influence on PEP implantation.

**Conclusion:**

Both suturing techniques were comparable regarding the need for EP-CPB or PEP implantation. Caution should be exercised when closing a large perimembraneous VSD with inlet extension.

**Table:** 

Abbreviations, acronyms & symbols	
ACC	= Aortic cross-clamp
CHB	= Complete heart block
CPB	= Cardiopulmonary bypass
PAB	= Pulmonary artery banding
PEP	= Permanent epicardial pacemaker
TVD	= Tricuspid valve detachment
VSD	= Ventricular septal defect

## INTRODUCTION

Ventricular septal defect (VSD) is the most frequent congenital heart pathology faced by congenital cardiac surgeons^[1-3]^. Despite increased theoretical and technical improvements, the bundle of His and its branches are almost always at risk of damage during surgical correction of VSD. The trajectory of the conduction system, which lies close to the posteroinferior rim of the VSD, may be damaged either by direct suturing or applying traction while exploring the VSD rim. While the reported incidence of surgically induced permanent complete heart block (CHB) after VSD closure remains between 0 and 8%^[1,2,4-11]^, the occurrence of iatrogenic CHB is expected to be less than 1% in which the occurrence has been mainly attributed to biological variations and lack of awareness of the disposition of the atrioventricular conduction axis^[1,9,12,13]^.

Although the suturing technique applied during patch closure of VSD was not described in detail in some reports^[1,4,7]^, a variety of suturing techniques have been implemented^[2,5,6,8-10,14,15]^. The aim of this retrospective study is to compare the continuous and combined suturing techniques in regards to the risk of permanent epicardial pacemaker (PEP) implantation in patients who underwent surgical VSD closure.

## METHODS

### Study Design and Population

This single centre survey was retrospectively designed. Patients who had consecutively undergone VSD closure between January 2006 and October 2015 were recorded in a database. Patients with an atrial septal defect, patent foramen ovale, pulmonary infundibular, valvar or supravalvar stenosis, patent ductus arteriosus and/or previous pulmonary artery banding (PAB) were included. Patients with an associated major cardiac anomaly (discordant atrioventricular or ventriculoarterial connection, double outlet right ventricle, tetralogy of Fallot, coarctation of the aorta, VSD with pulmonary atresia and atrioventricular septal defect) were excluded from the study. A total of 365 patients were included in the data analysis. The ethical committee of non-invasive clinical research of Ege University Faculty of Medicine (protocol: 15/9-4) approved the current retrospective survey on October 12, 2015.

### Study Variables

The demographic, clinical and operative characteristics of the patients were obtained from medical records. The type of VSD was determined according to the Congenital Heart Surgery Nomenclature and Database Project^[16]^. The size of the VSD was designated as large, moderate or small in accordance with the diameter of the aortic annulus. While transthoracic echocardiography was the primary diagnostic tool, catheterisation was utilised in select number of patients to estimate pulmonary vascular resistance (PVR) and perform pulmonary vascular reactivity test.

### Surgical Procedure

All surgical procedures were performed through median sternotomy under cardiopulmonary bypass (CPB) with selective bicaval cannulation and mild-to-moderate systemic hypothermia. Myocardial preservation was accomplished through intermittent cold blood cardioplegia. After examination of the location and borders of VSD, decision of whether to perform patch closure of VSD in continuous or combined fashion was made according to the surgeon's preference. None of the VSDs were closed with interrupted sutures all around. The distance between the muscle of Lancisi and the posteroinferior rim of the VSD was the main determinant for this decision. In continuous suturing, the patch closure of VSD was accomplished through continuous suturing all around the VSD while the suture bites at the posteroinferior rim of the VSD were shallow and located on the right ventricular side of the septum. In the combined technique, while the patch was anchored using pledgeted interrupted sutures corresponding to the posteroinferior rim of the VSD, the remaining rim of the VSD was sutured in continuous fashion. In both suturing techniques, the tricuspid side of the VSD patch was sewn close to the annulus of tricuspid septal leaflet in a continuous mattress fashion. In patients with pulmonary artery stenosis or previous PAB, pulmonary artery debanding and pulmonary artery reconstructions were performed while the aortic cross-clamp (ACC) was in place.

### Outcome Variables

PEP implantation was the primary outcome of the current study. The occurrence of CHB necessitating epicardial pacing at the time of weaning from CPB (EP-CPB) was also noted. Dexamethasone (0.5 mg/kg/day) was given to patients who required EP-CPB for at least 7 days. During in-hospital course, the postoperative day that the sinus rhythm was restored in patients who needed EP-CPB was also recorded.

### Statistical Analysis

Statistical analyses were performed using Statistical Package for Social Sciences (SPSS; IBM Corp., Armonk, NY, USA) version 19. None of the continuous variables showed a normal distribution, thus they were presented as median (range) values. For comparison, Mann-Whitney U-test, Pearson chi-square test or Fisher's exact test were used. A multivariable binary forward logistic regression analysis was performed to determine the covariates for EP-CPB and PEP implantation. A *P*-value of <0.05 was considered significant.

## RESULTS

### Population

**Fig. 1:**
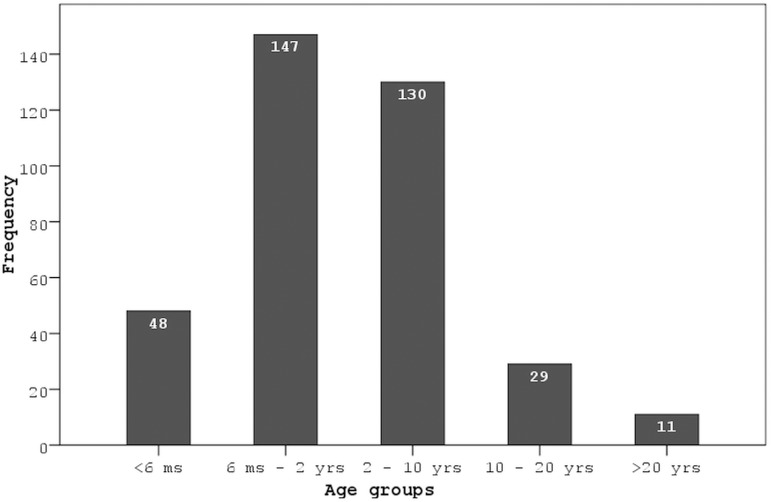
The distribution of age groups at the time of ventricular septal defect closure.

The median age and weight of the patients at the time of VSD closure were 15 months (range 27 days - 56.9 years) and 10 kg (range 3.5 - 100 kg), respectively. As shown in Figure 1, most of the patients (n=147, 40.3%) were aged between 6 months and 2 years. One hundred seventy-nine (49%) patients were male. There were 12 (3.3%) patients with chromosomal abnormality. The demographic and clinical characteristics of the patients are presented in Table 1. Apart from 10 (2.7%) patients with doubly-committed VSD, all VSDs were perimembraneous either with or without extension to the other segments of the interventricular septum. While perimembraneous VSD without extension was the most common (n=285, 78.1%) VSD characteristic, perimembraneous VSD with inlet extension (PM-I) was present in 44 (12.1%) patients. Eight (2.2%) patients had multiple VSDs. Excluding the patients with a history of PAB, there were 20 (5.5%) patients with pulmonary stenosis at various levels.

**Table 1:** The demographic and clinical characteristics of the patients.

Characteristics	Overall (n=365) n (%)	Continuous suturing (n=302) n (%)	Combined suturing (n=63) n (%)	*P*-value
Age, months	15 (27 days-56.9 years)*	22.3 (48 days-56.2 years)	12.7 (27 days-16.3 years)	0.002^α^
Weight, kg	10 (3.5-100)*	10.1 (4-100)	7.2 (3.5-46)	<0.001^α^
Male	179 (49)	155 (51.3)	24 (38.1)	0.056^β^
Previous pulmonary artery banding	63 (17.3)	46 (15.2)	17 (27)	0.025^β^
VSD type and characteristics				
PM	285 (78.1)	253 (83.8)	32 (50.8)	<0.001^β^
PM-I	44 (12.1)	18 (6)	26 (41.3)	<0.001^β^
PM-O	22 (6)	20 (6.6)	2 (3.2)	0.450^γ^
PM-IO	4 (1.1)	1 (0.3)	3 (4.8)	0.016^γ^
Doubly-committed	10 (2.7)	10 (3.3)	__	0.298^γ^
Multiple VSD^b^	8 (2.2)	5 (1.7)	3 (4.8)	0.290^γ^
VSD size				
Small	176 (48.2)	161 (53.3)	15 (23.8)	<0.001^β^
Moderate	114 (31.2)	85 (28.1)	29 (46)	0.005^β^
Large	75 (20.5)	56 (18.5)	19 (30.2)	0.038^β^
Catheterisation	140 (38.4)			
Mean pulmonary artery pressure, mmHg	35.5 (18-75)*	32 (18-75)	35 (19-70)	0.222^α^
Pulmonary vascular resistance, Wood	3.8 (1.8-9.0)*	4 (1.8-9)	3.3 (2-8)	0.682^α^
Qp/Qs	2.0 (1.6-3.0)*	2.1 (1.6-3.1)	2.1 (1.6-2.8)	0.971^α^
Tricuspid valve detachment	50 (13.7)	43 (14.2)	7 (11.1)	0.511^β^
ACC time, min*	36 (18-85)	35 (18-85)	45 (21-75)	<0.001^α^
CPB time, min*	50 (26-101)	47.5 (26-101)	58 (34-90)	<0.001^α^
ICU stay, days*	1 (1-20)	1 (1-16)	1 (1-20)	<0.001^α^
Hospital stay, days*	7 (2-34)	7 (2-34)	8 (4-25)	0.001^α^

ACC=aortic cross clamp; CPB=cardiopulmonary bypass; ICU=intensive care unit; PM=perimembraneous; PM-I=perimembraneous with inlet extension; PM-O=perimembraneous with inlet and outlet extension; PM-O=perimembraneous with outlet extension.

* Data are presented as the median (range). ^α^ Patients with previous pulmonary artery banding were not included. ^β^ Indicates an additional muscular VSD. ^α^ Mann-Whitney U-test, ^β^ Pearson chi-square test, ^γ^ Fisher's exact test.

There were 140 (38.4%) patients who were preoperatively evaluated by catheterisation to estimate PVR and assess pulmonary artery stenosis, for which the median age was 42.6 months (range 6.1 months - 56.9 years). The PVR was severely elevated (≥8 Wood) in five (3.6%) patients in whom the pulmonary vascular reactivity test was positive, thus none of the patients were considered inoperable.

### Surgery

VSD closure was accomplished through transatrial approach in the majority of the patients (95.1%). Other VSD approaches were performed through transatrial in five (1.4%) patients, transpulmonary in four (1.1%) and combined transatrial and transventricular in nine (2.5%). Continuous suturing (n=302, 82.7%) was the most frequently used technique. Fifty (13.7%) patients received tricuspid valve detachment (TVD), four of which required anteroseptal commissuroplasty to correct moderate tricuspid regurgitation following reattachment of the septal leaflet. While ASD/PFO closure (n=189, 51.8%) was the most frequent concurrent procedure, the other concurrent procedures were pulmonary artery debanding in 63 (17.3%) patients, PDA ligation/division in 27 (7.4%), pulmonary valvotomy in nine (2.5%), and right pulmonary artery reconstruction in three (0.8%). Pulmonary artery debanding in 63 (17.3%) patients was performed through supra-annular patch plasty of the main pulmonary artery using a Dacron patch. Interatrial communication in 12 patients was left open due to high PVR.

Because of the characteristics of the VSD, 12 patients required right ventriculotomy in which the VSD was doubly-committed in six, perimembraneous with outlet extension (PM-O) in three, and perimembraneous with both inlet and outlet extension (PM-IO) in three patients. In eight (2.2%) patients with multiple VSD, while three were closed with a separate patch, three were incorporated into the principal VSD patch, and two were closed via left ventriculotomy. The distribution of the VSD closure technique in terms of VSD characteristics are presented in Table 2. None of the patients were operated for recurrent VSD in the corresponding hospital admission. As revealed in Table 1, the rate of continuous suture technique increased significantly with increasing age and weight. While the proportion of continuous suturing was higher in perimembraneous VSD without extension, the rate of combined suture technique increased considerably with PM-I. Additionally, the rate of combined suture technique was higher in moderate and large VSDs. The ACC and CPB times, along with the duration of intensive care unit and hospital stay, were higher in patients who received combined suture technique.

**Table 2:** The distribution of surgical characteristics of ventricular septal defect closure according to VSD characteristics.

VSD characteristics	VSD approach				Tricuspid detachment (n=50)	Suturing technique	
	TA (n=347)	TV (n=5)	TA and TV (n=9)	TP (n=4)		Continuous (n=302)	Combined (n=63)
PM	283 (81.6)	__	2 (22.2)^α^	__	34 (68)	253 (83.8)	32 (50.8)
PM-I	44 (12.7)	__	__	__	10 (20)	18 (6)	26 (41.3)
PM-O	19 (5.5)	__	3 (33.3)	__	6 (12)	20 (6.6)	2 (3.2)
PM-IO	1 (0.3)	__	3 (33.3)	__	__	1 (0.3)	3 (4.8)
Doubly-committed	__	5 (100)	1 (11.1)	4 (100)	__	10 (3.3)	__

TA=transatrial; TP=transpulmonary; TV=transventricular

α Indicates patients with an additional separate muscular VSD-necessitated left ventriculotomy.

### Risk of Complete Heart Block

Overall, there were 25 (6.8%) patients with advanced second degree or CHB who needed EP-CPB. While the heart block recovered to sinus rhythm in 17 (68%) patients at a median of postoperative 5 days (range 1 - 11 days), PEP was implanted in eight (32%) patients (2.2% of all patients) at a median of postoperative 8 days (range 7 - 12 days). Neither EP-CPB nor PEP were required in patients with doubly-committed VSD, PM-O or PM-IO.

The univariate analysis for the need of EP-CPB and PEP is presented in Table 3. In patients who received EP-CPB, the proportion with either a small or large VSD was lower than those who did not receive EP-CPB (24% *vs.* 76%, *P*=0.012; 40% *vs.* 60%, *P*=0.013, respectively). In contrast, the proportion of patients with large VSD was higher in those who received PEP (75% *vs.* 25%, *P*=0.001). The proportion of patients with perimembraneous VSD without extension was higher in those who needed EP-CPB (60% *vs.* 40%, *P*=0.024) but in contrast, it was lower in patients who received PEP implantation (37.5% *vs.* 62.5%, *P*=0.018). On the other hand, while the rate of patients with PM-I was significantly lower than that of patients who needed EP-CPB (40% *vs.* 60%, *P*<0.001), it was substantially higher in patients who received PEP (62.5% *vs.* 37.5%, *P*<0.001). As presented in Table 4, the comparison of the suturing techniques according to the need for EP-CPB and PEP implantation among patients with perimembraneous VSD without extension and in patients with PM-I revealed no statistically significant difference.

**Table 3:** Univariate analysis of the influence of independent variables on requirement for epicardial pacing at the time of weaning from cardiopulmonary bypass and permanent epicardial pacing.

Variable		EP-CPB			PEP		
		Yes (n = 25) n (%)	No (n = 340) n (%)	*P*-value	Yes (n = 8) n (%)	No (n = 357) n (%)	*P*-value
Age, days*		401 (27-7011)	575.5 (48-20518)	0.052^α^	649.5 (180-2754)	551 (27-20518)	0.959^α^
Weight, kg*		8.5 (4-69)	10 (3.5-100)	0.052^α^	9 (7-18)	10 (3.5-100)	0.945^α^
Male		12 (48)	167 (49.1)	0.914^β^	2 (25)	177 (49.6)	0.309^γ^
VSD size							
Small	Yes	6 (24)	170 (50)	0.012^β^	1 (12.5)	175 (49)	0.092^γ^
	No	19 (76)	170 (50)		7 (87.5)	182 (51)	
Moderate	Yes	9 (36)	105 (30.9)	0.594^β^	1 (12.5)	113 (31.7)	0.441^γ^
	No	16 (64)	235 (69.1)		7 (87.5)	244 (68.3)	
Large	Yes	10 (40)	65 (19.1)	0.013^β^	6 (75)	69 (19.3)	0.001^γ^
	No	15 (60)	275(80.9)		2 (25)	288 (80.7)	
VSD characteristics							
PM	Yes	15 (60)	270 (79.4)	0.024^β^	3 (37.5)	282 (79)	0.018^γ^
	No	10 (40)	70 (20.6)		5 (62.5)	75 (21)	
PM-I	Yes	10 (40)	34 (10)	<0.001^γ^	5 (62.5)	39 (10.9)	<0.001^γ^
	No	15 (60)	306 (90)		3 (37.5)	318 (89.1)	
Multiple VSD	Yes	2 (8)	6 (1.8)	0.178^γ^	__	8 (2.2)	1.0^γ^
	No	23 (92)	334 (98.2)		8 (100)	349 (97.8)	
Previous PAB		5 (20)	58 (17.1)	0.919^γ^	2 (25)	61 (17.1)	0.910^γ^
VSD approach							
Transatrial		25 (100)	322 (94.7)	0.483^γ^	8 (100)	339 (95)	1.0^γ^
Transventricular		__	5 (1.5)	1.0^γ^	__	5 (1.4)	1.0^γ^
Transatrial and transventricular		__	9 (2.6)	0.876^γ^	__	9 (2.5)	1.0^γ^
Tricuspid valve detachment		5 (20)	45 (13.2)	0.517^γ^	1 (12.5)	49 (13.7)	1.0^γ^
Suturing technique							
Continuous		18 (72)	284 (83.5)	0.231^γ^	7 (87.5)	295 (82.6)	1.0^γ^
Combined		7 (28)	56 (16.5)		1 (12.5)	62 (17.4)	

PAB=pulmonary artery banding.

* Data are presented as the median (range).

α Mann-Whitney U-test,

β Pearson chi-square test,

γ Fisher's exact test.

**Table 4:** Comparison of suturing techniques according to the need for epicardial pacing at the time of weaning from cardiopulmonary bypass and permanent epicardial pacing in perimembraneous VSD and perimembraneous VSD with inlet extension.

VSD type		EP-CPB (n=25) n (%)			PEP (n=8) n (%)		
		Yes	No	*P*-value	Yes	No	*P*-value
PM	Continuous	11 (73.3)	242 (89.6)	0.127^γ^	3 (100)	250 (88.7)	1.0^γ^
	Combined	4 (26.7)	28 (10.4)		__	32 (11.3)	
PM-I	Continuous	7 (70)	11 (32.4)	0.078^γ^	4 (80)	14 (35.9)	0.160^γ^
	Combined	3 (30)	23 (67.6)		1 (20)	25 (64.1)	

γ Fisher's exact test.

In multivariate analysis, PM-I was found to be the significant covariate associated with the need for both EP-CPB (OR=6.0; 95% CI [2.5, 14.4]; *P*<0.001) and PEP implantation (OR=9.02; 95% CI [1.97, 41.36]; *P*=0.005) (Table 5). The large VSD was another significant covariate for PEP implantation (OR=8.63; 95% CI [1.63, 45.8]; *P*<0.011).

**Table 5:** Multivariate analysis for the need for permanent epicardial pacemaker implantation.

Variable	EP-CPB			PEP		
	Exp(B)	95% CI	*P*-value	Exp(B)	95% CI	*P*-value
PM-I	6.0	2.50, 14.40	<0.001	9.02	1.97, 41.36	0.005
Large VSD	-	-	-	8.63	1.63, 45.80	0.011

The independent variables included the suture technique, large VSD, multiple VSD, tricuspid valve detachment, perimembraneous VSD without extension, perimembraneous VSD with inlet extension, transatrial approach, aortic cross-clamp and cardiopulmonary bypass time

## DISCUSSION

The reported prevalence of iatrogenic permanent CHB after isolated VSD closure remains between 0 and 8%^[2,4-9,11,15]^. In a study that comprised 828 patients who underwent VSD closure surgery, the rate of EP-CPB was reported to be 7.7%^[11]^. In that study, while the rhythm returned to sinus in 48 patients, 16 (1.9%) patients required PEP implantation. Furthermore, a body weight less than 4 kg and inlet VSD were identified as risk factors for the development of permanent CHB. In the current study, PM-I (*P*<0.001) was also found to be a risk factor for the development of CHB, both in the univariate and multivariate analyses. On the other hand, weight was not found to have an influence on the need for either EP-CPB or PEP implantation. The main reason for this lack of association may be related to the low number of patients weighing less than 4 kg in the current study, because PAB has been still the procedure of choice in some centres to avoid the consequences of open heart surgery in low-weight patients^[17]^.

Biventricular performance diminishes soon after congenital heart surgery due to either the impact of CPB, changes in the loading conditions of the ventricles, or attachment of an akinetic patch to the ventricular septum itself during VSD closure^[17-20]^. Quinn et al.^[19]^ reported a decrease in fractional segmental shortening localised to the interventricular septum in patients who underwent VSD closure surgery. During the interrupted suture technique, with the use of a patch larger than VSD size results in a larger segment of the septum to be compromised^[19,20]^. While not evaluated in the current study, the avoidance of ventricular dysfunction soon after VSD closure was the main reason why interrupted suture closure surrounding a VSD was not utilised.

Shallow stitching at the posteroinferior rim of the VSD during the continuous suture technique (<1.5 mm depth and <4 mm away from the rim) was found to be an important factor for avoiding postoperative CHB^[8,21]^, which was supported in the current series. To the best of our knowledge, only few articles in the literature have compared the type of suture technique in regards to the development of CHB^[15]^. Continuous and interrupted suturing techniques were compared in a cohort of 231 patients, and CHB developed in five patients who received the continuous suture technique^[15]^. An interesting finding of the present study was the shift in significance in terms of the need for EP-CPB. While it was higher in patients with perimembraneous VSD without extension, the rate of PEP was greater in patients with PM-I (Tables 3 and 4). These inverted changes in the rates were strongly related to the recovery of the sinus rhythm in patients with perimembraneous VSD without extension, which, in our opinion, was a strong indicator of the importance of the tractions and manipulations applied to obtain better exposure of the VSD rims. Another important point of the current study was the importance of the inlet extension of a VSD for the development of heart block, and neither of the suturing techniques applied were found to have a significant influence in either the univariate or multivariate analyses. Among 4432 patients with surgical perimembraneous VSD closure enrolled in the Pediatric Cardiac Care Consortium Database, Down syndrome was reported to be the most significant risk factor associated with PEP implantation, indicating the importance of inlet extension of a perimembraneous VSD^[7]^. From this point of view, the application of continuous suturing in patients with inlet extension is the most striking finding of the current study, which, in our opinion, was the reason underlying the higher prevalence of CHB necessitating PEP implantation when compared to that described in the literature. Because the bundle of His runs through the thin rim of the muscle separating the VSD from the tricuspid valve annulus^[3,21]^. On the other hand, CHB was not expected to occur in a VSD with inlet extension when the combined suture technique was applied. From our point of view, this issue could have been addressed using a larger patch which may have been able to keep sufficiently away from the conduction system.

When compared to the interrupted suture technique, the ACC and CPB times have been reported to be significantly reduced with continuous suture closure of a VSD^[15]^. As revealed in Table 1, the ACC and CPB times were considerably lower in patients in whom the continuous suture technique was utilised. The reduction in ACC and CPB times gained through applying continuous suture closure of a VSD may represent the most favourable advantage of this technique.

Additionally, continuous suture closure of a VSD was reported to be a risk factor for the development of a residual VSD^[15]^. The durability of the posteroinferior rim where the shallow continuous sutures were placed was the primary concern for the development of a residual VSD. The current study demonstrated that neither the continuous nor the combined suture closure had an unfavourable influence on the development of residual VSD necessitating reoperation.

With the aim of improving visualisation of a VSD, TVD was performed in a considerable proportion of the patients who underwent VSD closure operation^[6,12,14,22]^. Additionally, TVD has not been identified as a risk factor for the development of CHB^[6,12,14]^. TVD was utilised in 13.7% of patients in the current study. In support of the literature, TVD in the current series was not found to be a risk factor for EP-CPB or PEP implantation.

Aside from the retrospective design of this study, the major limitation was that it did not assess VSD closure by the interrupted suture technique completely surrounding the VSD^[15]^. Thus, this commonly utilised technique could not be compared with the techniques applied in the current study. The literature describes a significant number of patients who received PEP implantation in whom the rhythm returned to sinus over a variety of periods^[7,11,13]^. Thus, another important limitation of the current study was that the out-hospital course of the patients was not assessed.

## CONCLUSION

In conclusion, based on the improved theoretical and technical advancements, VSD closure can be safely and efficiently performed with a very low rate of CHB. The current study showed that there was no difference between the suture techniques regarding the development of permanent CHB, which put forward the biological variations and the manoeuvres applied to obtain better exposure during surgery as the primary cause of the development of heart block. Development of CHB after VSD closure occurred independent of the performance of TVD, which can be safely utilised in patients with multiple chordal attachments that interrupt the visibility of the VSD rims. Additionally, although they seem to have a favourable influence on reducing ACC and CPB times, surgeons should be more cautious or avoid the continuous suturing technique when closing a PM-I.

**Table:** 

Authors' roles & responsibilities	
MFA	Substantial contributions to the conception or design of the work; agreement to be accountable for all aspects of the work in ensuring that questions related to the accuracy or integrity of any part of the work are appropriately investigated and resolved; final approval of the version to be published
EŞ	Substantial contributions to the conception or design of the work; drafting the work or revising it critically for important intellectual content; agreement to be accountable for all aspects of the work in ensuring that questions related to the accuracy or integrity of any part of the work are appropriately investigated and resolved; final approval of the version to be published
MD	Substantial contributions to the conception or design of the work; agreement to be accountable for all aspects of the work in ensuring that questions related to the accuracy or integrity of any part of the work are appropriately investigated and resolved; final approval of the version to be published
YÖŞ	Substantial contributions to the conception or design of the work; drafting the work or revising it critically for important intellectual content; final approval of the version to be published
HŞ	Substantial contributions to the conception or design of the work; agreement to be accountable for all aspects of the work in ensuring that questions related to the accuracy or integrity of any part of the work are appropriately investigated and resolved; final approval of the version to be published
REL	Substantial contributions to the conception or design of the work; drafting the work or revising it critically for important intellectual content; final approval of the version to be published
YA	Substantial contributions to the conception or design of the work; drafting the work or revising it critically for important intellectual content; final approval of the version to be published
